# Development and Validation of a New Assessment Tool for Simulation-Based Training in Ultrasound-Guided Peripheral Venous Catheterization

**DOI:** 10.7759/cureus.101992

**Published:** 2026-01-21

**Authors:** Jacob Linnet, Ebbe Thinggaard, Mikkel Scavenius, Lene Russell, Kim Ekelund, Magnús Pétur B Obinah

**Affiliations:** 1 Department of Emergency Medicine, Nordsjaellands Hospital, Hillerød, DNK; 2 Copenhagen Academy for Medical Education and Simulation, Copenhagen University Hospital, Copenhagen, DNK; 3 Department of Gynecology, Copenhagen University Hospital - Rigshospitalet, Copenhagen, DNK; 4 Department of Pediatric and Obstetric Anesthesia, Copenhagen University Hospital - Rigshospitalet, Copenhagen, DNK; 5 Department of Anesthesiology and Intensive Care, Copenhagen University Hospital - Herlev and Gentofte, Copenhagen, DNK; 6 Department of Anesthesiology and Intensive Care, Copenhagen University Hospital – Rigshospitalet, Copenhagen, DNK; 7 Department of Breast and Plastic Surgery, Zealand University Hospital - Roskilde, Roskilde, DNK

**Keywords:** difficult vascular access, novel assessment tool, peripheral vein catheter placement, simulation trainer, ultrasound-guided

## Abstract

Introduction

Establishing intravenous access is a common clinical procedure, yet peripheral venous catheter placement can be challenging in certain patient populations. Using ultrasound improves success rates but remains operator-dependent and requires training. Simulation-based training enhances procedural skills without risk to patients but requires a standardized measure of the acquired skills. This study presents and validates a new assessment tool for simulation-based training in ultrasound-guided peripheral venous catheterization, enabling safe transition from simulation to clinical implementation.

Methods

This exploratory, rater-blinded study recruited participants from two Danish university hospitals. Participants were allocated to one of two groups according to experience level. Each participant performed three ultrasound-guided peripheral venous catheter placements on a phantom. All attempts were video recorded and rated by two blinded raters using the new assessment tool. Validity evidence was examined using Messick’s framework, focusing on content validity, response process, internal structure, relationships to other variables, and consequences of testing.

Results

Fifty participants were enrolled, of whom 46 were included in the final analysis. Internal consistency was high, with an intraclass correlation coefficient (ICC) of 0.85. The tool effectively discriminated between novices and experienced participants (p < 0.001). A pass/fail score of 18 points was established, with a false-negative rate of 4.5% and a false-positive rate of 12.5%.

Conclusion

We found the new assessment tool valid and reliable, effectively distinguishing novices from experienced participants and setting a passing cutoff score. This supports its use in simulation-based training to standardize competence evaluation, serving as a gateway to subsequent assessment in clinical practice.

## Introduction

Establishing intravenous access in patients is a common hospital practice [1,2]. Peripheral vein catheter placement is typically based on palpation and visual inspection of a target vein, but in some patients, successful placement can be challenging [3].

It is estimated that 10% of admitted patients fail to get a peripheral vein catheter, despite at least two attempts by an experienced clinician [3]. This may cause delayed treatment and increased discomfort due to multiple cannulation attempts, and in some cases, repeated failures may require placement of a central venous catheter [2].

Ultrasound-guided peripheral vein catheter placement increases success rates [4-7], increases patient satisfaction, and reduces the number of central venous catheter placements [8,9]. Yet ultrasound-based techniques are operator-dependent and require training in skills, which can hinder general certification and implementation [10,11].

Simulation-based training has been shown to improve technical skills in a range of procedures [12-14] by allowing novices to practice technical skills on simulators or specialized phantoms [15]. However, implementation of simulation-based training requires a validated assessment tool to establish a minimum passing standard of proficiency, thereby ensuring a certain level of competence before performing the procedure on patients [15].

Assessment tools supported by validity evidence are necessary, as they enable standardized evaluation and certification. Several checklists and rating tools for competence assessment in ultrasound-guided peripheral vein catheter placement have been described [16,17]. However, validity evidence has only been explored for one rating scale, and this was in a clinical setting [18]. To our knowledge, no studies have examined the validity evidence of an assessment tool for ultrasound-guided peripheral vein catheter placement in a simulation-based setting. 

The aim of this study was to develop a simple and easy-to-use assessment tool for ultrasound-guided peripheral vein catheter placement and to explore its validity evidence for simulation-based training.

## Materials and methods

Study design

This was an exploratory, rater-blinded study conducted at Copenhagen Academy for Medical Education and Simulation and two university hospitals (Zealand University Hospital and Copenhagen University Hospital) in Copenhagen, Denmark. Two groups of medical staff with different experience levels performed ultrasound-guided venous access on a phantom with vessels mimicking veins and with echogenic properties.

As recommended by the American Educational Research Association [19], Messick’s framework [20] was used to assess the validity supporting the new assessment tool. This framework is based on the evidence from five sources: content, internal structure, response process, relation to other variables, and consequences of testing.

Investigation of validity

Content validity investigates if the content reflects the underlying construct of interest. We based the construct of the procedure on a previous Delphi study [21], which investigated the consensus of content among experts in ultrasound-guided peripheral vein catheter placement. Furthermore, a multidisciplinary expert panel was established to subsequently evaluate and tailor this content to fit a simulation-based training setting.

The Response Process refers to the evidence of data integrity, meaning that all sources of error associated with test administration are controlled or eliminated to the maximum extent possible. This was addressed by randomization and blinding of raters. Raters underwent rater training prior to assessment, setup was standardized, new phantoms were used for each participant, and all video recordings were collected by the corresponding author. Internal structure investigates whether the test score is reliable. This was analyzed by calculating the intraclass correlation coefficient (ICC) with absolute agreement to assess the degree of agreement between raters.

The relation to other variables examines the extent to which a task correlates with other variables, such as experience level. This was assessed by comparing novice and experienced participants’ scores to evaluate whether the tool could discriminate based on experience level.

Consequences of testing examines the implications of score-based decisions. We used the contrasting group method [22] to establish a pass/fail cut-score and evaluated the rates of false positive results and false negative results.

Test setup and materials

**Figure 1:**
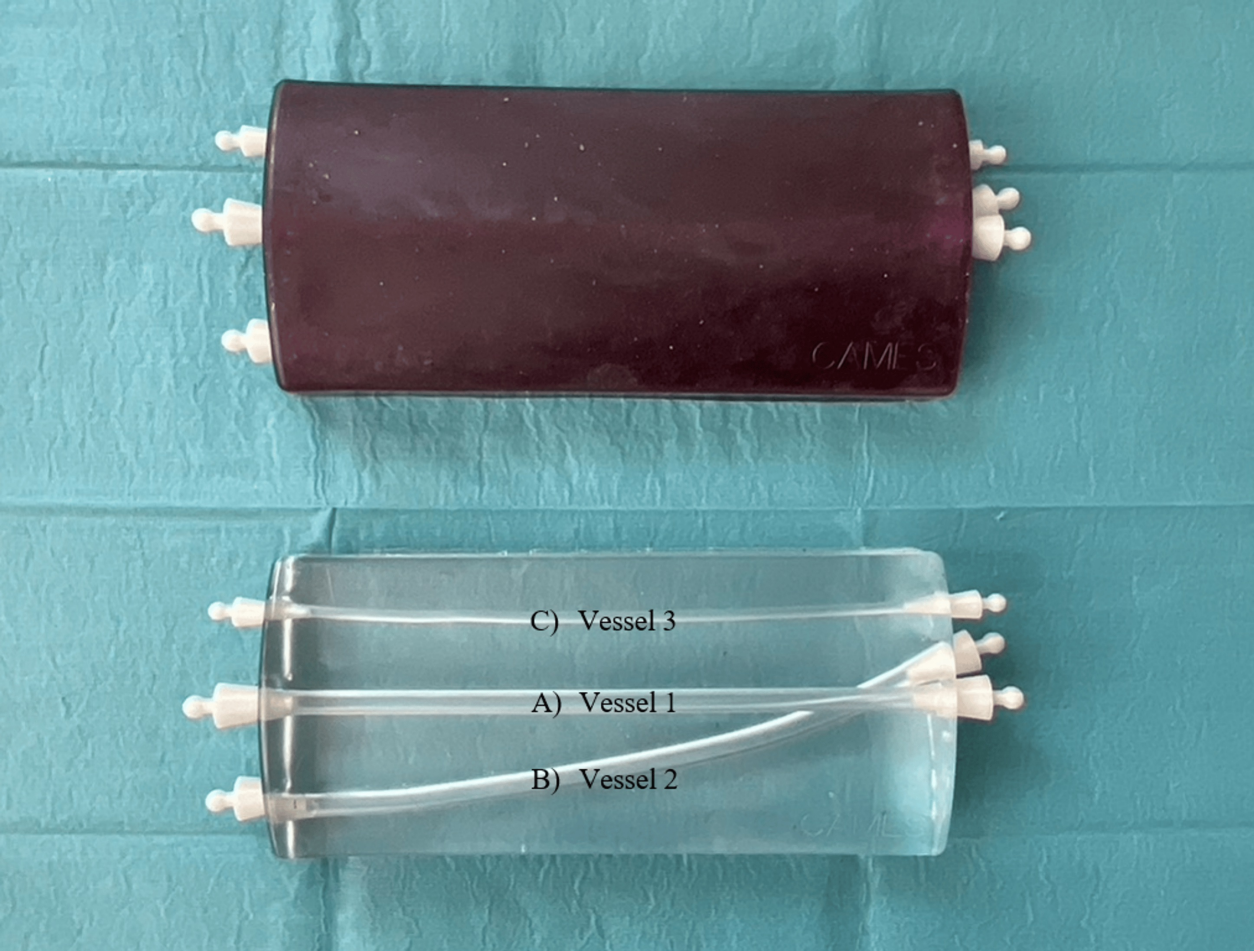
Phantom for assessment A) Vessel 1: A large, straight, superficial vessel (Ø6 mm); B) Vessel 2: A large, curved, deep vessel (Ø6 mm); C) Vessel 3: A small, straight, superficial vessel (Ø3 mm)

**Figure 2:**
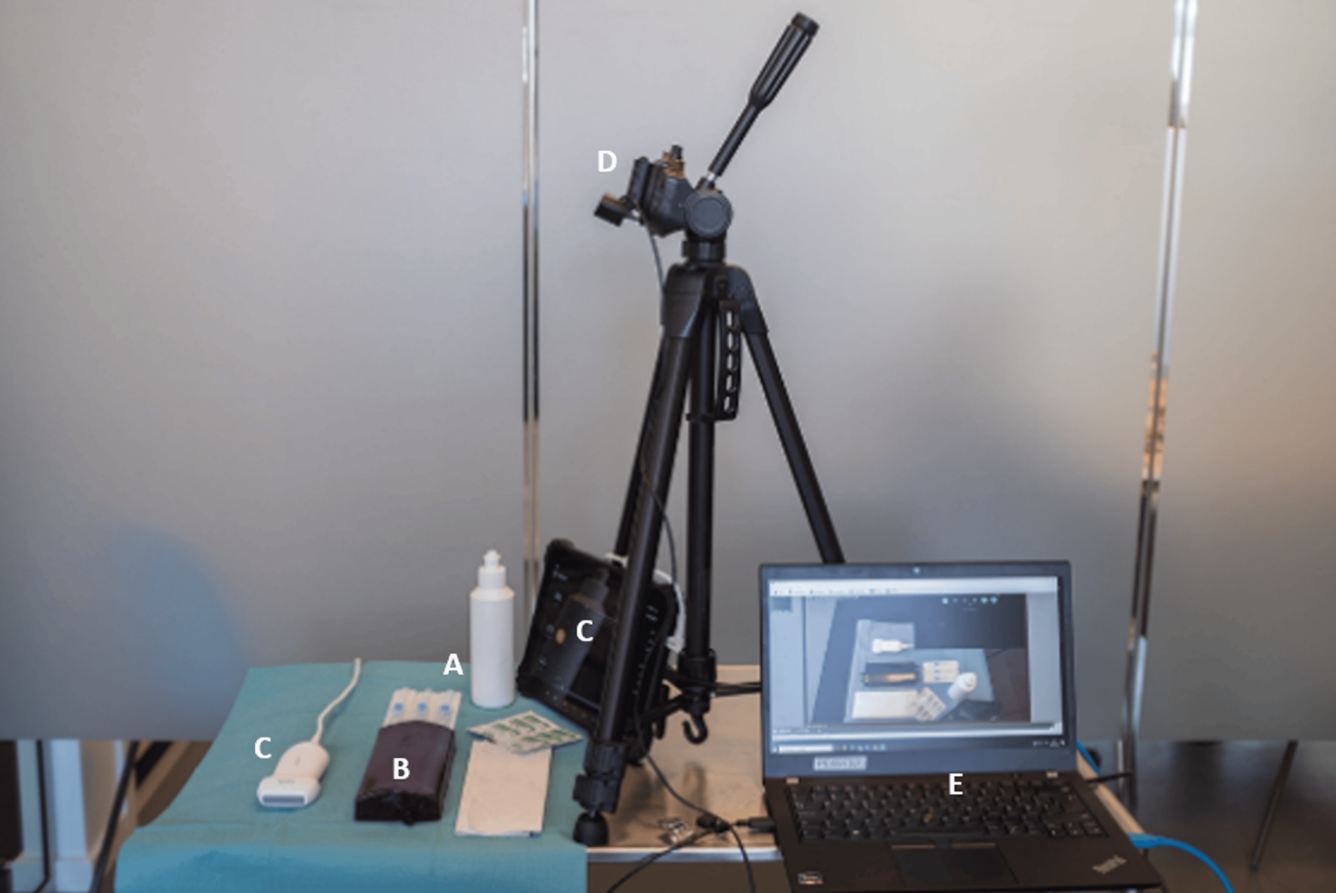
Test setup and materials A) Remedies for peripheral vein catheter insertion and ultrasound gel; B) Phantom; C) Tablet with ultrasound image and high-frequency ultrasound probe; D) Video camera; E) Software for synchronizing the two video feeds

We used a previously described low-cost, open-access phantom (Figure 1) shown to have a high fidelity [23]. Ultrasound was performed using a Lumify L12-4 Android probe, Philips Futurepad 10, and the Philips Lumify Ultrasound App (Philips Privacy Office, Amsterdam, the Netherlands). Furthermore, standard remedies for peripheral vein catheter placement and ultrasound were used. The test setup and materials are depicted in Figure 2.

Participants

The study included two groups: a novice group and an experienced group. The inclusion criteria for the novice group were 1) at least one year of experience performing peripheral vein catheter placement and 2) no experience placing peripheral vein catheters using ultrasound. Novices were recruited among nurses and medical students. The inclusion criteria for the experienced participants were at least one year of experience placing peripheral vein catheters using ultrasound. Experienced participants were recruited among anesthesiologist specialists and trainees. All included participants completed a demographic questionnaire (Supplemental Material 1).

Assignment

Participants were shown a two-minute introduction video (Video 1) demonstrating a short-axis/out-of-plane ultrasound-guided placement of a catheter in vessel 1 of the phantom. Participants were then asked to place a catheter in each of the phantom vessels, using the technique demonstrated in the video, with the following order: vessel 1, vessel 2, vessel 3. No time limit was set for the assignment. 

**Video 1:** Introduction video

Video recording

Each catheter placement attempt was recorded by joining two separate video feeds (synchronizing software: iSpyConnect v7.2.1.0, iSpyConnect, Margaret River, Australia), one showing the procedure field (video recorder: USB Webcam Pro, Sandberg A/S, Birkerød, Denmark) and one showing the ultrasonography screen. The procedure field recording was set up to include only the gloved hands of the participants to preserve pseudo-anonymity. All video filenames were randomized with a computer-generated ID in order to blind raters and thereby minimize the halo-effect risk [24].

Rating

Two raters with clinical experience in placing peripheral vein catheters with ultrasound assessed participant videos separately using the created assessment tool. Ratings were shared with the corresponding author for analysis.

Performance on vessel 2 was selected for the final assessment, as this vessel, with depth and curvature, most accurately simulated a normal peripheral vein suitable for ultrasound-guided placement. However, performance was also rated on vessels 1 and 3 in case either of these vessels provided better discrimination between novices and experts than the anticipated vessel 2.

Prior to video assessment, both raters underwent rater training. This involved assessing six pilot videos created specifically to demonstrate different levels of performance. The raters' results were discussed and compared to ensure alignment.

Statistical analysis

In addition to the analyses described in the investigation of validity section, we determined discrimination between results in the two groups using an independent sample t-test. A two-sided p-value < 0.05 was considered significant. Linear correlation was assessed by computing Pearson correlation coefficients to analyze performance scores and experience between the two groups. Statistical analysis was performed using IBM SPSS Statistics for Windows, version 28 (IBM Corp., Armonk, NY, USA).

Ethical consideration

As the study did not involve patients, no ethical approval was required according to Danish law. Written informed consent was obtained from all participants, who could withdraw at any time during the study period (Supplemental Material 2).

## Results

**Figure 3:**
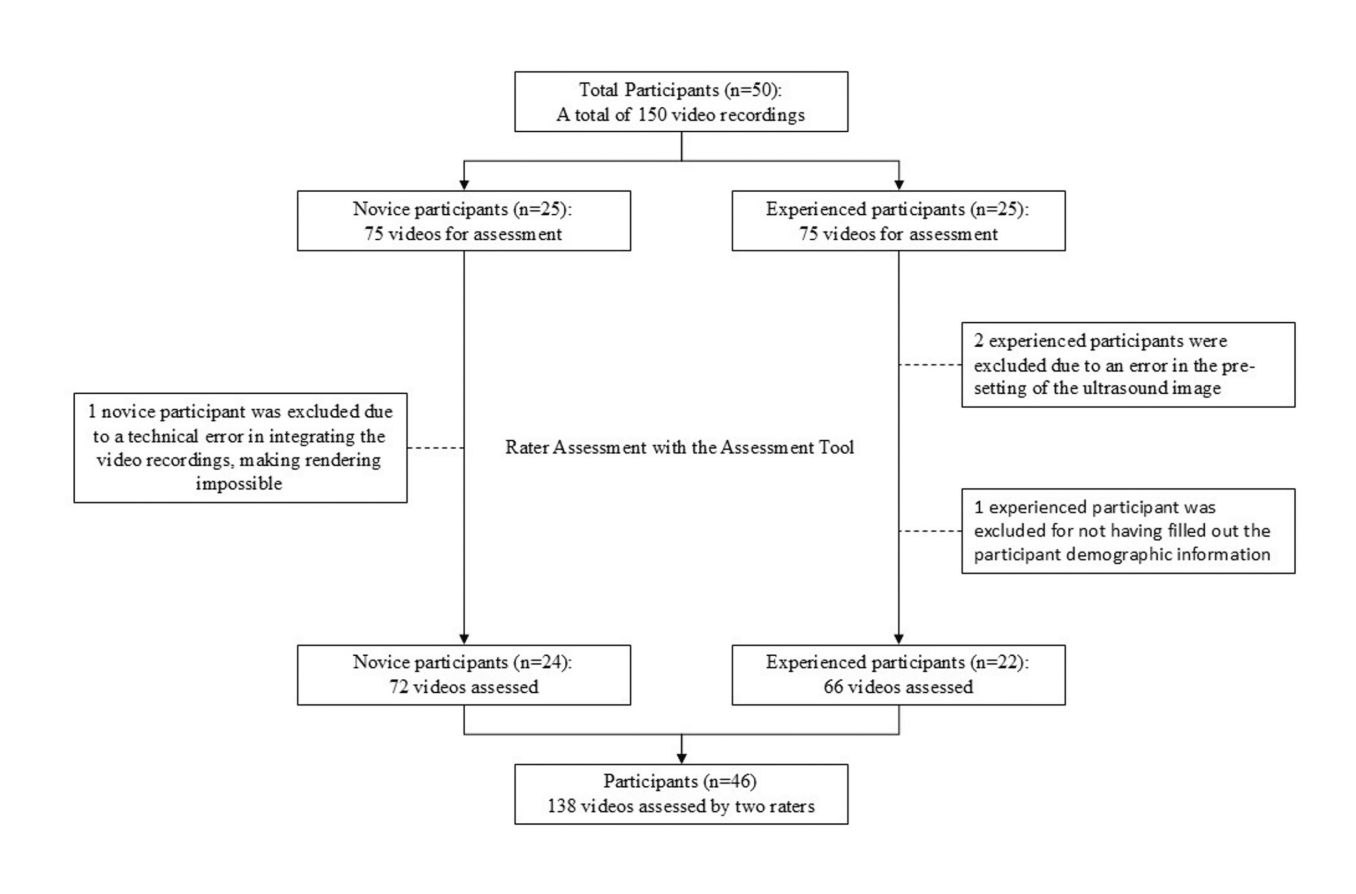
Flowchart of participant inclusion and assessment process

Fifty participants participated in the study and completed all three vascular access attempts. Four participants were excluded due to errors in the inclusion process or errors in the recorded videos (Figure 3).

The final statistical analysis was based on 138 videos obtained from 46 participants, consisting of 22 experienced participants and 24 novice participants (Table 1).

**Table 1:** Participant demography PGY: post-graduate year; PVC: peripheral venous catheter

Participants	Groups	
-	Experienced	Novice
Gender (n=46)	-	-
Male	11 (50%)	5 (20.8%)
Female	11 (50%)	19 (79.2%)
Hospital (n=46)	-	-
Copenhagen University Hospital	4 (18.2%)	9 (37.5%)
Zealand University Hospital	18 (81.8%)	15 (62.5%)
Doctors (n=22)	-	-
PGY 1-2	0 (0%)	-
PGY 3-5	6 (27.3%)	-
PGY 6-8	4 (18.2%)	-
PGY 8<	12 (54.5%)	-
Nurses (n=20)	-	-
PGY 1	-	1 (4.2%)
PGY 2	-	1 (4.2%)
PGY 3-5	-	2 (8.3%)
PGY 5-8	-	3 (12.5%)
PGY 8<	-	13 (54.2%)
Medical students (n=4)	-	-
Uncategorized	-	4 (16.7%)
Hand dominance (n=46)	-	-
Right	20 (90%)	18 (75%)
Left	2 (9%)	6 (25%)
Experience in PVC placement (years)	-	-
Mean (minimum; maximum)	14 (3;40)	13 (1;35)
Experience in ultrasound-guided peripheral vein catheter placement (years)	-	-
Mean (minimum; maximum)	7 (1;20)	-

Data were assessed as normally distributed. Content validity was investigated through our multidisciplinary expert panel consisting of anesthesiologists and a medical educational researcher. After several meetings in the multidisciplinary panel, items and verbal anchors from the Delphi study [21] were selected, discussed, modified, adjusted, and rejected so that content reflected the underlying construct of interest for assessment in a simulation-based environment. The resulting assessment tool adhered to a five-point Likert format, with five items and verbal anchors at one, three, and five points (Table 2).

**Table 2:** The Easy Access Assessment Tool PVC: peripheral venous catheter

Item	Points (verbal anchor)				
Ultrasound image optimization	1 (Fails to optimize the image)	2	3 (Competent image optimization attempted)	4	5 (Excellent image optimization)
Sanitizing	1 (The area around the puncture site is not sanitized during PVC placement)	2	3 (The area around the puncture site is inadequately sanitized, or contamination occurs during PVC placement)	4	5 (The area around the puncture site is sanitized and kept sanitized during PVC placement)
Needle cannulation	1 (More than one perforation attempts were performed throughout the procedure)	2	3 (One perforation attempt was performed, but with hesitation)	4	5 (One perforation attempt was performed with a smooth, purposeful movement)
Securing catheter	1 (The PVC needle tip is not visualized in the vessel)	2	3 (The PVC is secured with 1-2 needle tip visualizations in the vessel)	4	5 (The PVC is secured with 3 or more needle tip visualizations in the vessel)
Saline flush	1 (The PVC is not flushed with saline)	2	3 (The PVC is unsuccessfully flushed with saline)	4	5 (The PVC is successfully flushed with saline)

**Figure 4:**
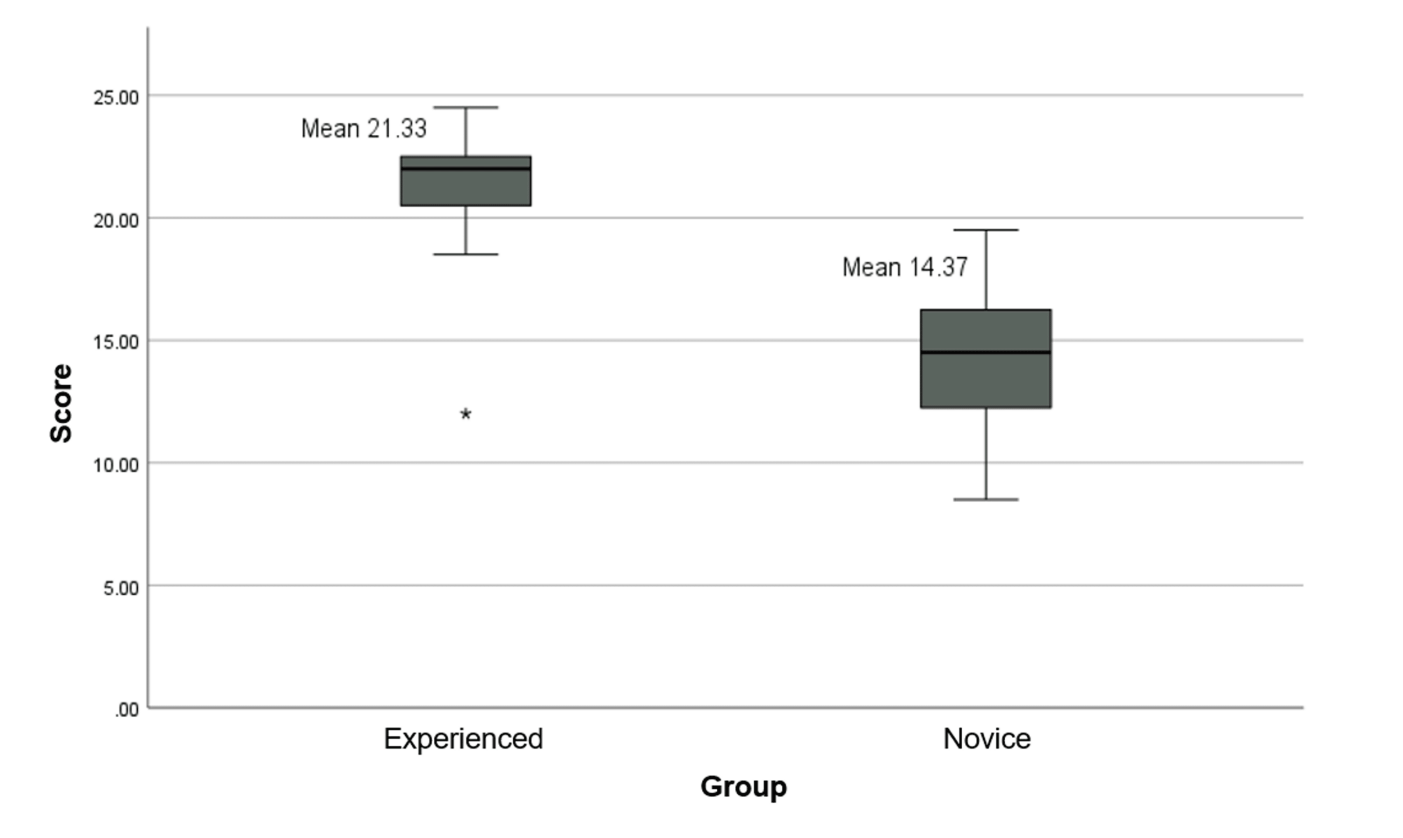
Boxplot of performance scores in the novice and experienced groups

The ICC, calculated to evaluate internal structure, was 0.85 (95% CI: 0.60-0.90, p < 0.001). In relation to other variables, the scores differed significantly between groups (p < 0.001), with novices having a mean score of 14.4 and experienced participants a mean score of 21.3 (Figure 4).

**Figure 5:**
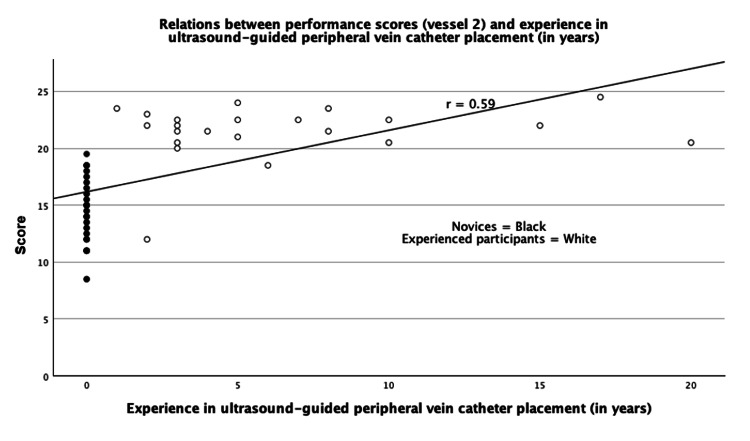
Pearson's correlation coefficient Figure 5 illustrates the relationship between years of experience in ultrasound-guided peripheral vein catheter placement and performance score among participants. The Pearson correlation coefficient was r = 0.59.

The Pearson correlation coefficient between years of clinical experience with ultrasound-guided peripheral vein catheter placement and performance score in vessel 2 was r = 0.59 (Figure 5).

**Figure 6:**
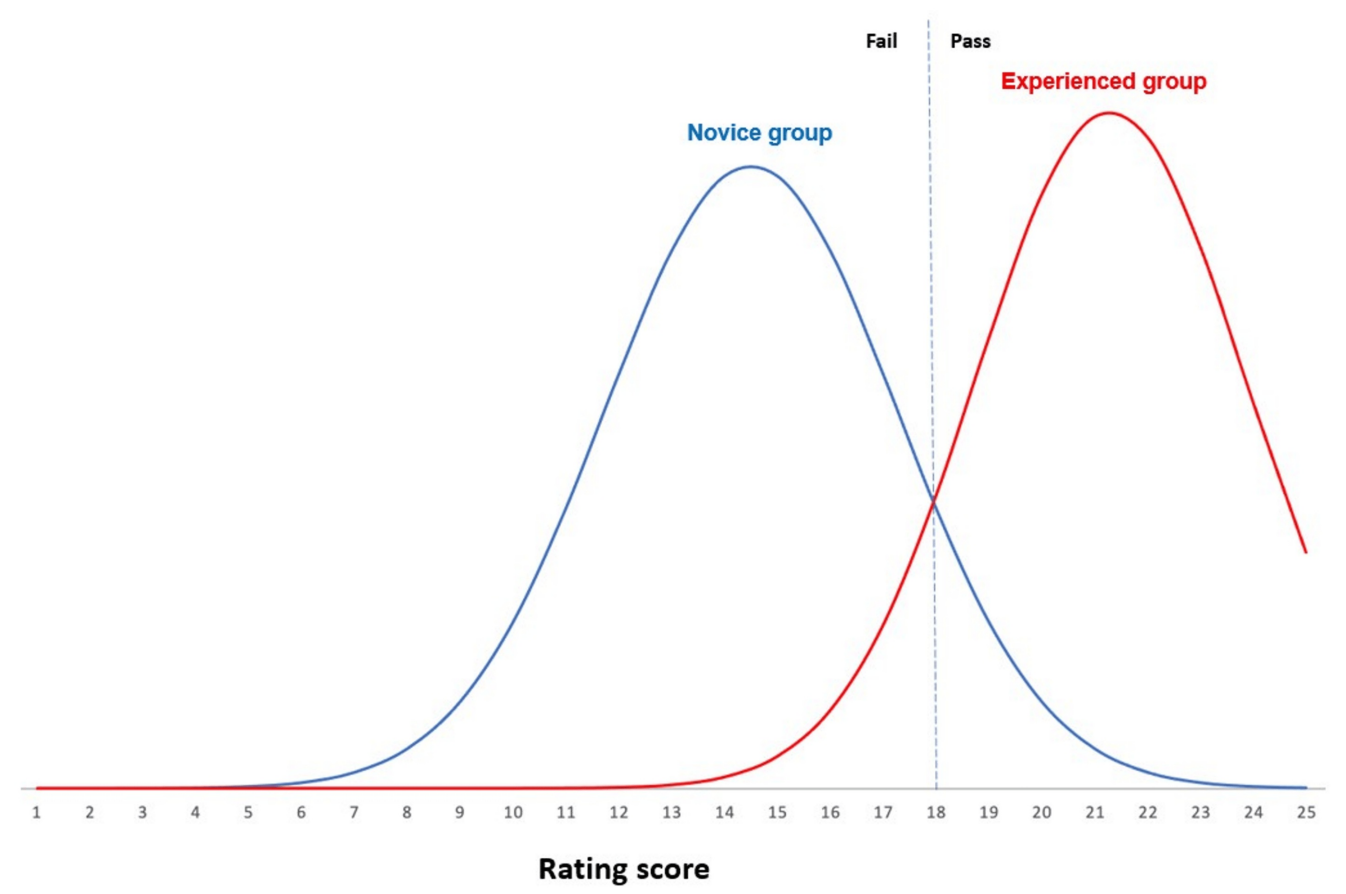
Determination of the pass/fail cutoff score This figure shows the assumed normal distribution of scores in the novice and experienced groups based on the means and standard deviations obtained from vessel 2. The pass/fail score was determined at the intersection between the two distributions using the contrasting groups method [22], resulting in a cut-off level of 18 points.

Using the contrasting group method [22] to evaluate the consequences of testing, we found a pass/fail cut-score of 18 points, resulting in a false negative rate of 4.5% and a false positive rate of 12.5% (Figure 6).

The validity investigation is summarized in Table 3.

**Table 3:** Summary of validity evidence ICC: intraclass correlation coefficient

Source of validity evidence	Validity evidence for the assessment tool
Content validity	The assessment tool is aligned with the construct, though a final assessment tool with five items based on a Delphi consensus study and evaluation by a multidisciplinary expert panel.
Response process	The test setup was standardized, and all data were collected by the same author. New and unmarked phantoms were used for all participants. Participants' videos were randomized and blinded for rating. Raters went through rater training prior to the assessment.
Internal structure	Inter-rater reliability was high in vessel 2 (ICC=0.85).
Relation to other variables	Novices (mean 14.4) and experienced participants (mean 21.3) scored significantly differently (P<0.0001) and with a moderate Pearson correlation r=0.59.
Consequences of testing	A pass/fail score of 18 results in a false negative rate of 4.5% and a false positive rate of 12.5%.

## Discussion

We developed and validated an assessment tool using Messick's validity framework [20]. The tool discriminates between experience levels, establishes a reliable pass/fail standard, and is compatible with a low-cost open-access phantom [23] and ultrasound equipment, enabling widespread implementation.

High internal structure, i.e., inter-rater reliability, is crucial for assessment tools, as rater inconsistency is one of the greatest threats to reproducibility [25]. Our tool demonstrated high internal structure (ICC=0.85), making it acceptable for moderate-stakes assessments [25]. While larger item lists tend to have higher ICC, ultrasound-guided peripheral vein catheter placement is a relatively simple procedure, making it difficult to obtain a large item list without compromising content validity [25]. Placing peripheral vein catheters with ultrasound has relatively few complications for patients with difficult vascular access compared to the landmark technique, and we believe it is well-suited for moderate-stakes assessments.

The assessment tool shows the ability to discriminate between novices and experienced practitioners with a Pearson correlation of 0.59, indicating a moderate positive correlation between experience and performance score. This moderate correlation could reflect rapid skill acquisition and early performance plateaus in this procedure. Such a tendency is also illustrated by the Pearson correlation graph (Figure 5), where experienced participants demonstrated similar performance scores regardless of their years of experience.

The established pass/fail cut score resulted in a false negative rate of 4.5% (experienced participants failing) and a false positive rate of 12.5% (novice participants passing). It is noted that two of the three novices who received a passing score were medical students. This finding may be coincidental or may reflect potential bias, perhaps due to heterogeneity within the novice group or prior ultrasound experience. Determining the significance of this finding is beyond the scope of this study due to the population size and because the questionnaire did not address other ultrasound experiences other than for peripheral vein catheterization.

As noted in two previous systematic reviews [16,17], while numerous checklists and rating tools for competence assessment have been documented, only the Peripheral Ultrasound-Guided Vascular Access Rating Scale described by Primdahl et al. [18] is evidence-based and validated for assessing competence in ultrasound-guided peripheral vein catheter placement. This assessment tool, while based on a global rating scale and Messick's contemporary framework [20], like ours, was designed to assess the performance of patients, and the validity evidence was therefore explored only in a clinical setting and not in a simulation-based setting. Our tool, supported by validity evidence, is therefore the first to establish a minimum passing standard for ultrasound-guided peripheral vein catheter placement in a simulation-based environment. This ensures that trainees will achieve adequate proficiency in the simulation center before transitioning to clinical application of the acquired skills.

When comparing our results to the assessment tool developed by Primdahl et al. [18], we find similarity in ICC, false negative, and false positive rates, but a lower correlation between experience and performance score. However, our study had a high number of participants [26], and our analysis was based on a very high number of assessed recordings, whereas Primdahl et al. included fewer participants (n=15) and recordings (n=42).

We believe that this new and validated assessment tool, combined with an inexpensive phantom that is freely available for reproduction online [23], provides a practical and scalable solution that can help standardize preclinical simulation-based training in ultrasound-guided peripheral vein catheter placement. In a consensus-based educational curriculum, this tool would help enable competence in ultrasound-guided peripheral vein catheter placement and support a safe and better transition from simulation-based training to supervised assessment in clinical practice.

Strength and limitations

This study has some limitations. The questionnaire did not assess other ultrasound experience beyond ultrasound-guided peripheral vein catheter placement; this may represent a potential bias in the assessment of procedural competence. Rater drift was not formally assessed during the study period. Given the short duration of the study, the potential impact of rater drift is expected to be limited. The dichotomous classification of participants into only two groups leaves out distinguishing intermediates from experts. Because the procedure is associated with rapid skill progression, an intermediate phase is expected to be brief, making inclusion of such a group challenging. The novice group included nurses and medical students, resulting in a more heterogeneous group compared with the experienced group. However, all included novices met the same inclusion criteria.

The main strength of this study is the blinded ratings to minimize the halo effect bias. The halo effect is an important source of bias that should be minimized to maintain data integrity, as onsite rating tends to yield higher scores due to a potential assessor-trainee relationship [27]. Assessment tools relying on subjective rater scores are particularly vulnerable to this bias, since blinded assessment can be impractical in educational settings compared to onsite rating. 

Perspectives

Propagating certification and competence in ultrasound-guided peripheral vein catheter placement remains challenging due to the resources required for healthcare professionals to achieve proficiency. However, broad implementation can help patient satisfaction and reduce the need for central venous catheter placements, and tools supported by validity evidence, like ours, are important for creating standardized pathways from simulation-based training to clinical practice and implementation.

Skills acquired in simulation-based training are not necessarily transferred directly to the clinical environment, as shown by systematic reviews [28]. A study investigating the transferability, i.e., the correlation between scores obtained with this assessment tool and clinical performance could, therefore, provide valuable additional insights. If such a study showed acceptable transfer of skill, it would support the use of this tool, consensus- and evidence-based training curriculum for ultrasound-guided peripheral vein catheter placement [16,17].

## Conclusions

This study demonstrates that the Easy Access Assessment Tool is valid and reliable for evaluating performance in ultrasound-guided peripheral vein catheter placement in a simulation-based setting. A cutoff score of 18 points was identified, representing a reference level of experienced performance. These findings support the use of the tool in pre-clinical simulation-based training, serving as a standardized gateway to subsequent assessment in clinical practice.

## Appendices

Supplemental material 1

Participant Demographic Questionnaire Sheet

**Table 4:** Questionnaire sheet Credits: Jacob Linnet

General information	Answer
Name	-
Participant number	-
Hospital	-
Department	-
Profession/specialty	-
Educational Level (KBU, Intro, HU, Specialized)	-
Post-graduate year (PGY)	-
Hand dominance (Right, left, or both)	-
Experience in PVC placement (in years)	-
Experience in USG-PVC placement (in years)	-
Sex	-

Supplemental material 2 

Consent Form in Danish 

Erklæring fra forsøgsperson:

Jeg har fået skriftlig og mundtlig information, og jeg ved nok om formål, metode, fordele og ulemper til at sige ja til at deltage. Jeg ved, at det er frivilligt at deltage, og at jeg altid kan trække mit samtykke tilbage. Jeg giver samtykke til at deltage i forskningsprojektet, og jeg har fået en kopi af dette samtykkeark, samt en kopi af den skriftlige information om projektet, til eget brug.

Deltagers Navn:   ________________________________________ Deltager nr:          ________________________________________

Dato:                    ________________________________________

Underskrift:          ________________________________________

Erklæring fra den, der afgiver information:

Jeg erklærer, at forsøgspersonen har modtaget mundtlig og skriftlig information om forsøget.

Efter min overbevisning er der givet tilstrækkelig information til, at der kan træffes beslutning om deltagelse i forsøget.

Navnet på den, der har afgivet information: __________________________________

Dato: ____________________________   

Underskrift: _________________________________

English Translation of the Consent Form

Statement from the Participant: I have received written and oral information, and I know enough about the purpose, methods, benefits, and disadvantages to agree to participate. I understand that participation is voluntary, and that I may withdraw my consent at any time. I hereby consent to participate in the research project, and I have received a copy of this consent form as well as a copy of the written information about the project for my own use.

Participants Name: ________________________________________

Participant No.: ________________________________________

Date: ________________________________________

Signature: ________________________________________

Statement from the Person Providing the Information:

I declare that the participant has received oral and written information about the study. In my view, sufficient information has been given to allow the participant to make a decision regarding participation.

Name of the person providing the information: __________________________________

Date: ________________________________________

Signature: ________________________________________
